# Thermostabilization of inactivated polio vaccine in PLGA-based microspheres for pulsatile release

**DOI:** 10.1016/j.jconrel.2016.05.012

**Published:** 2016-07-10

**Authors:** Stephany Y. Tzeng, Rohiverth Guarecuco, Kevin J. McHugh, Sviatlana Rose, Evan M. Rosenberg, Yingying Zeng, Robert Langer, Ana Jaklenec

**Affiliations:** David H. Koch Institute for Integrative Cancer Research, Massachusetts Institute of Technology, Cambridge, MA 02139, USA

**Keywords:** Drug delivery, Vaccine delivery, Single-administration vaccines, Controlled release kinetics, Vaccine stability

## Abstract

Vaccines are a critical clinical tool in preventing illness and death due to infectious diseases and are regularly administered to children and adults across the globe. In order to obtain full protection from many vaccines, an individual needs to receive multiple doses over the course of months. However, vaccine administration in developing countries is limited by the difficulty in consistently delivering a second or third dose, and some vaccines, including the inactivated polio vaccine (IPV), must be injected more than once for efficacy. In addition, IPV does not remain stable over time at elevated temperatures, such as those it would encounter over time in the body if it were to be injected as a single-administration vaccine. In this manuscript, we describe microspheres composed of poly(lactic-*co*-glycolic acid) (PLGA) that can encapsulate IPV along with stabilizing excipients and release immunogenic IPV over the course of several weeks. Additionally, pH-sensitive, cationic dopants such as Eudragit E polymer caused clinically relevant amounts of stable IPV release upon degradation of the PLGA matrix. Specifically, IPV was released in two separate bursts, mimicking the delivery of two boluses approximately one month apart. In one of our top formulations, 1.4, 1.1, and 1.2 doses of the IPV serotype 1, 2, and 3, respectively, were released within the first few days from 50 mg of particles. During the delayed, second burst, 0.5, 0.8, and 0.6 doses of each serotype, respectively, were released; thus, 50 mg of these particles released approximately two clinical doses spaced a month apart. Immunization of rats with the leading microsphere formulation showed more robust and long-lasting humoral immune response compared to a single bolus injection and was statistically non-inferior from two bolus injections spaced 1 month apart. By minimizing the number of administrations of a vaccine, such as IPV, this technology can serve as a tool to aid in the eradication of polio and other infectious diseases for the improvement of global health.

## Graphical abstract

**Figure:**
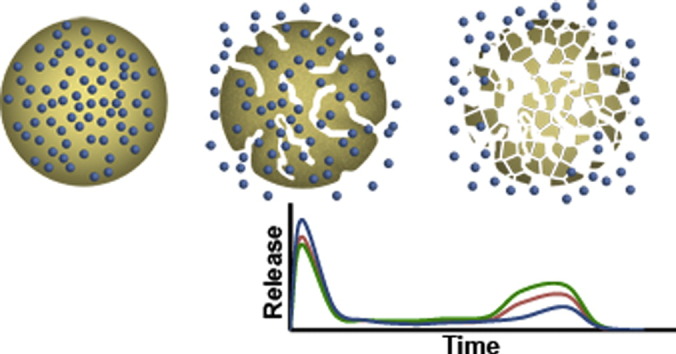

## 1 Introduction

Vaccines have been essential in combating infectious diseases in both children and adults. However, for many vaccines, multiple doses must be administered over the course of months, which can be challenging in developing countries where vaccine administration is limited by patient access. For example, among incompletely vaccinated children, > 40% receive at least one dose of the diphtheria-tetanus-pertussis vaccine but do not receive all three recommended doses, therefore remaining unprotected [1]. In order to achieve better vaccination coverage, especially in the developing world, the World Health Organization (WHO) Expanded Program for Immunization (EPI) suggests that an ideal vaccine should be heat-stable, require only a single shot, and be easy to administer [2]. The requirements for stability and single administration are challenging for many vaccines, such as the poliovirus vaccine, which has been shown to have very poor thermal stability [3]. The two types of polio vaccines currently being used are the oral polio vaccine (OPV), a live attenuated virus, and the inactivated polio vaccine (IPV). IPV, while much safer than OPV with no risk of reversion to a disease-causing virus [4], must be injected more than once for efficacy [5]. This can be prohibitive to protection and eradication efforts, as studies in some regions of the world have found that coverage for a second dose of vaccines is much lower than that for the first dose [6]. Therefore, a formulation that can deliver the equivalent of two or more boluses of a vaccine with only a single administration would be very valuable for disease eradication [2].

However, single administration of vaccines and especially IPV is challenging because of its poor thermostability [7]. In general, IPV is administered in the D-antigen conformation, which is used to evaluate clinical formulations and is characteristic of infective viruses; over time at high temperature, it converts to the C-antigen conformation, which does not confer protective immunity. If injected only once into a patient and retained long-term in a local depot, IPV would need to retain its D-antigenicity and ability to elicit a protective immune response after incubation inside the body at 37 °C for several months in the presence of various biological factors as well as the depot material. Work has been done on identifying various stresses that affect IPV stability [8], screening excipients that will stabilize IPV through drying and storage conditions [9], [10], and improving the immunogenicity of IPV through the use of adjuvants [11]. However, these studies do not address the fact that multiple injections are required for protection.

Controlled release microspheres are a promising technology for single-injection vaccines such as IPV. However, to our knowledge, no studies have previously been published on controlled release of IPV from injectable, biodegradable microspheres. Biocompatible, degradable microspheres can be injected once and remain in the body as a depot, with degradation of the material controlling the timing of vaccine release [12], [13]. Poly(lactic-*co*-glycolic acid) (PLGA) is among the most well-studied materials for microsphere formulations. As a result of its biocompatibility, PLGA has been a component of a number of FDA-approved devices [14], [15]. In certain cases, large, hydrophilic macromolecules, such as proteins and particularly virus particles such as IPV, can be designed to be released from PLGA particles immediately in an initial burst of release, followed by slow or no release while the polymer begins to degrade by bulk erosion, and finally a second burst of accelerated release [16], [17], [18]. Moreover, extended presentation of antigens after injection, as would be expected from a depot of injected microspheres, has been shown to improve the immune response compared to a bolus [19]. Design of PLGA microspheres to exhibit pulsatile release could potentially lead to even better response, as it would mimic the 2–3 injections currently used in the clinic [12].

Despite their promise as a tool for disease eradication, PLGA microparticle-based systems present many points at which IPV can be destabilized during the formulation process and over the course of release at body temperature. Moreover, IPV is composed of three serotypes of inactivated poliovirus, each of which may respond differently to stresses. Various excipients, including carbohydrates, amino acids, and salts have been studied as potential stabilizers for IPV, with a combination of a sugar or polyol, monosodium glutamate (MSG), and magnesium chloride (MgCl_2_) greatly increasing IPV thermostability in the dry state [9]. Here, we study various excipients from these classes to assess their ability to protect the stability of all three IPV serotypes during processing, drying, and incubation in a wet and 37 °C environment over time. We further use a water-in-oil-in-oil (w/o_1_/o_2_) double emulsion method in order to co-encapsulate these small-molecule excipients with IPV in PLGA microspheres with minimal initial leakage [20]. We then assessed their efficacy in stabilizing IPV in PLGA microspheres and use a polycationic dopant, Eudragit E, to modulate PLGA degradation as well as buffering the acidic PLGA degradation products, thereby achieving multiple bursts of IPV release over time. Finally, microspheres composed of PLGA and Eudragit E, encapsulating IPV and stabilizing excipients, were injected intramuscularly into rats to show non-inferior humoral immune response compared to bolus injections spaced 1 month apart.

## 2 Materials and methods

### 2.1 Materials

Trivalent inactivated polio vaccine (tIPV) stocks were provided by Statens Serum Institut (SSI; Copenhagen, Denmark) with starting concentrations of 327 DU/mL, 70 DU/mL, and 279 DU/mL of type 1, 2, and 3, respectively (Brünhilde strain type 1, MEF-1 strain type 2, and Saukett strain type 3). D-antigen content of samples was assessed using the monoclonal D-antigen enzyme-linked immunosorbent assay (ELISA) kit purchased from SSI. Poly(d,l-lactic-*co*-glycolic acid) (PLGA) with average molecular weight and lactide-to-glycolide ratios of 12 kDa and 50:50 (PLGA502H) and Eudragit® E PO (EPO) were purchased from Evonik (Essen, Germany). Gelatin from cold water fish skin, d-sorbitol, d-sucrose, d-trehalose, maltodextrin, monosodium glutamate (MSG), magnesium chloride hexahydrate (MgCl_2_), magnesium hydroxide (Mg(OH)_2_), heavy mineral oil, and the surfactant Span® 80 were purchased from Sigma Aldrich (St. Louis, MO). AlexaFluor® 680 succinimidyl ester was from Life Technologies (Carlsbad, CA). Serum binding antibody titers were measured by indirect ELISA using monoclonal mouse antibodies specific for D-antigenic type 1 (HYB295-15-02), type 2 (HYB294-06-02), or type 3 (HYB300-05-02) poliovirus from Thermo Scientific Pierce (Life Technologies Corporation, Grand Island, NY) and polyclonal goat anti-rat IgG antibody conjugated with horse radish peroxidase (HRP) from Abcam (Cambridge, MA). SigmaFast OPD (*o*-phenylene diamine) peroxidase substrate was purchased from Sigma.

### 2.2 IPV thermostability studies

To assess stability of IPV after incubation at 37 °C, tIPV was concentrated using 0.5-mL Amicon® Ultracel® centrifugal filters (Merck Millipore Ltd., Billerica, MA) with 100-kDa molecular weight cutoff. Each filter was blocked with 2.5% solution of gelatin by centrifugation at 14,000 rcf for 10 min and then removal of the gelatin retentate from the filter. In 0.5-mL increments, 2 mL of tIPV stock solution was concentrated by centrifuging at 14,000 rcf for 10 min. Following concentration, the IPV retentate was diluted with 0.5 mL sterile distilled water and concentrated once more in order to remove the small-molecule components in the tIPV medium. Using this method, 2 mL tIPV stock was concentrated to 19 ± 1 μL with 148-, 150-, and 145-fold increase in type 1, 2, and 3 concentration, respectively, from the tIPV stock concentration (Supplementary Fig. S1), and this will be referred to hereafter as IPV_conc_.

The IPV_conc_ was then diluted to 16 DU/mL, 3.4 DU/mL, and 13.6 DU/mL type 1, 2, and 3, respectively, in sterile distilled water. This dilute IPV solution was then mixed 1:1 (v/v) with a sterile solution of an excipient or excipient mixture in water (see Table 1). The final IPV concentration in all groups, assuming no loss of D-antigenicity during the filtration and concentration processes, was 8 DU/mL, 1.7 DU/mL, and 6.8 DU/mL type 1, 2, and 3, respectively. Because lower IPV concentrations were found to be more difficult to stabilize (Supplementary Fig. S2), excipient screening was carried out at a relatively low IPV concentration, corresponding to approximately ten-fold lower concentration than the normal concentration used for clinical administration (80 DU/mL, 16 DU/mL, and 64 DU/mL). IPV/excipient mixtures were then aliquoted into sealed vials and stored at 37 °C on a tube revolver. At predetermined time points, some aliquots were removed and analyzed by ELISA. All results were normalized to the starting D-antigen content of the formulation before storage at 37 °C.

### 2.3 IPV processing stability studies: water-in-oil emulsion

IPV stability was assessed after exposure to aqueous/organic solvent interfaces and the physical mixing processes used to form water-in-oil emulsions. Nineteen microliters of IPV_conc_ was mixed with 10 μL of water or aqueous solution of excipients at a 100:1 excipient:IPV mass ratio, and the mixture was added to a solution of 25 mg PLGA502H in 1 mL dichloromethane. These were emulsified *via* sonication on ice at 20% amplitude for 30 s. Two milliliters of ELISA sample dilution buffer (1 × PBS with 1% TritonX-100, 1% bovine serum albumin [BSA], and 0.001% phenol red) was added to the emulsion and mixed well by vortexing. The organic dichloromethane layer was then evaporated over 3 h of stirring, and the remaining aqueous phase was analyzed for D-antigen content by ELISA.

### 2.4 IPV processing stability studies: vacuum-drying

Ten microliters water or aqueous solution of excipients was added to 19 μL of IPV_conc_ using a 100:1 excipient:IPV mass ratio. This final volume was placed in a vacuum for 1 h at room temperature to simulate the final drying of microspheres. The dried product was then redissolved in ELISA sample dilution buffer and tested *via* ELISA. Percent recovery was calculated by normalizing the D-antigen measurement to that of a formulation prepared in the same way but left liquid rather than drying under vacuum.

### 2.5 IPV pH-sensitivity studies

Salt solutions of varying pH were prepared. Because of differences in the IPV-stabilizing properties of different salts, 1 × PBS was used for all solutions, with small amounts of 1 M hydrochloric acid (HCl) or 1 M sodium hydroxide (NaOH), rather than other buffers optimized for very high or low pH. tIPV was diluted 1:200 in solutions of pH 1, 4.5, 6, 7.4, 8, or 9 and stored at either 4 °C or 37 °C. After 7 days, the dilute IPV solutions were neutralized with either HCl or NaOH, and D-antigen recovery was measured *via* ELISA.

### 2.6 Emulsion microsphere formulations

A double emulsion method was used to encapsulate IPV along with stabilizing excipients in PLGA-based microspheres. Because many of the excipients used are salts and other small molecules, a water-in-oil-in-oil (*w*/*o*_1_/*o*_2_) method was used to minimize the diffusion of excipients out of the particles during the formulation process. The aqueous (*w*) phase consisted of 2 mL tIPV, concentrated to form 19 μL of IPV_conc_ and mixed with 10 μL of a solution of excipient(s) in water. All excipients and excipient combinations tested are listed in Table 2. The first oil (*o*_1_) phase was prepared by dissolving 25 mg PLGA502H in 1 mL dichloromethane (DCM). In some formulations, EPO was also dissolved in the *o*_1_ phase. For all formulations, the tIPV was 0.2% of the final formulation by mass. The second oil phase (*o*_2_) consisted of heavy mineral oil with 3% (v/v) Span® 80 surfactant.

To form the first emulsion, the *w* phase was added to the *o*_1_ phase, and the mixture was sonicated at 20% amplitude for 30 s. For formulations containing Mg(OH)_2_, solid Mg(OH)_2_ was first dispersed in the *o*_1_ phase by sonication before adding *w* and sonicating again. To form the second emulsion, 1 mL *o*_2_ was added to the *w*/*o*_1_ emulsion and vortexed for 5 s at 3500 rpm. This *w*/*o*_1_/*o*_2_ was then poured into stirring heavy mineral oil (0.4% final surfactant concentration). The entire suspension was stirred for 3 h at room temperature to allow complete DCM extraction and evaporation. Particles were collected by centrifugation at 200 rcf for 3 min at 4 °C, washed three times with hexanes, and dried for 1 h under vacuum at room temperature. All DCM was expected to have evaporated after 3 h stirring during microsphere formulation [21], [22], aided in part by its high solubility with the mineral oil continuous phase. All hexanes, which were present only on external surfaces of the particles, were vaporized upon exposure to high vacuum during drying. The dry microspheres were either resuspended in buffer for immediate use or stored dry at 4 °C with desiccant until use.

### 2.7 pH of the PLGA microenvironment

The pH and release kinetics of PLGA particles was assessed simultaneously. For release kinetics, IPV was labeled with AlexaFluor® 680 (AF680) for measurement of total IPV release by fluorescence detection. tIPV stock was concentrated to IPV_conc_ as described above using centrifugal filters. The final product was washed three times with 0.1 M carbonate/bicarbonate buffer (pH 8.9) and collected into a tube. AF680 succinimidyl ester dye was dissolved in DMSO at 5 mg/mL and added to the IPV suspended in carbonate/bicarbonate buffer, for a final dye:IPV molar ratio of approximately 300:1. The resulting mixture was protected from light and incubated at room temperature for 1 h on a tube revolver. Free dye was removed from the reaction using a PD-10 pre-packed column with Sephadex G-25 beads, equilibrated with a 9:1 (v/v) water-to-DMSO mixture. The product was then purified again in a PD-10 column equilibrated with water alone. This final product (AF680-IPV) was lyophilized overnight. Before use, the dry powder was resuspended in water to the same starting volume as IPV_conc_ and was used at the same concentration to make emulsion microspheres. All formulations containing AF680-IPV were kept out of direct light. Labeling efficiency of the fluorophore was assessed *via* NanoDrop (Thermo Scientific) by measuring the absorbance of the protein-dye conjugate at 679 nm (AF680 absorbance peak, molar extinction coefficient *ε* = 184,000 cm^− 1^ M^− 1^) and 280 nm (protein absorbance peak, estimated *ε* = 5580 cm^− 1^ M^− 1^). AF680-IPV with a 76:1 molar ratio of dye:virion was used. Similar procedures were used to label BSA (AF680-BSA), with a final dye:protein molar ratio of 3:1.

For release studies, microspheres containing AF680-IPV were resuspended at 10 mg/mL in release buffer containing 1 × PBS with 0.2% BSA and 0.02% sodium azide. Tubes containing the microparticle suspension were incubated at 37 °C on a tube revolver. At predetermined time points, tubes of particles were removed from the incubator and centrifuged at 1500 rcf at 4 °C for 5 min. The particles in each tube were resuspended in release buffer, vortexed, and returned to 37 °C until the next time point. Supernantants were measured in a black-walled, clear-bottom 96-well plate using an Infinity® M1000 Pro microplate reader (Tecan, Mannëdorf, Switzerland), with excitation/emission of 679 nm/702 nm, respectively. The pH of the supernant was also measured using pH strips and reported as the average pH value measured for all the samples ± standard deviation.

### 2.8 Release studies for unlabeled IPV-containing microspheres

IPV microspheres were resuspended at 10–15 mg/mL in release buffer containing 1 × PBS with 50 mM HEPES, 0.2% BSA, 0.001% phenol red, and 0.02% sodium azide. The same buffer was used for all microsphere formulations. Tubes containing the microparticle suspension were incubated at 37 °C on a tube revolver. At predetermined time points, tubes of particles were removed from the incubator and centrifuged at 1500 rcf at 4 °C for 5 min. The supernatant of each tube was removed and stored at 4 °C for up to 1 week before analysis. The particles in each tube were resuspended in release buffer, vortexed, and returned to 37 °C until the next time point. D-antigen in the collected supernatants was measured *via* ELISA.

### 2.9 Microsphere characterization

The amount of IPV encapsulated in microspheres was measured using a method similar to that reported by Kim et al. [23]. Five milligrams of microspheres was dissolved in 1 mL of DCM. After the polymer had dissolved, leaving small amounts of organic-insoluble solids, the suspension was centrifuged at 15,000 rcf for 15 min, and the polymer-containing DCM solution was removed, similar to the method described by. The precipitate was washed three times with DCM to ensure complete removal of the water-insoluble polymer(s). Residual DCM was removed from the precipitate by vacuum. The precipitate, which included IPV, was then dissolved in 1 mL of 1 × PBS. The concentration of IPV in the aqueous fraction was measured by ELISA using polyclonal antibodies isolated from rabbits immunized with denatured IPV (see Supplementary methods). Three (n = 3) samples were measured for each microsphere batch, and encapsulation efficiency is reported as mean ± standard deviation.

Microspheres were imaged with scanning electron microscopy (SEM). Micrographs were obtained using a FEI XL30 FEG Environmental SEM with backscatter detector. Microsphere size was measured using a Multisizer 3 Coulter Counter (Beckman Coulter).

### 2.10 Immune response to IPV in rodents

Female Wistar rats aged 8–12 weeks were anaesthetized with isoflurane and immunized by intramuscular (IM) injection in the hind quadriceps. Experimental groups tested were free IPV as a single bolus; free IPV as two boluses, spaced four weeks apart; controlled-released microspheres containing encapsulated IPV and stabilizing excipients; and free IPV as a single bolus alongside blank microspheres containing only stabilizing excipients. For microsphere groups, the particles were resuspended in saline using a method similar to those previously reported [24], [25] before injection. For all groups, the same total D-antigen IPV (24 DU type 1, 4.8 DU type 2, and 19.2 DU type 3) was injected. The amount of IPV in microspheres was calculated from the total D-antigen IPV released *in vitro*, as measured by ELISA. All groups included n = 10 animals. Blood was collected from the lateral tail vein of all animals at 2 week intervals. The whole blood was clotted, and the serum was separated *via* centrifugation at 2000 rcf for 10 min at 4 °C and kept frozen at − 20 °C until analysis. For negative control, two untreated rats of the same strain and age were exsanguinated and their serum pooled.

To measure IPV-specific binding antibody (IgG) titers, monoclonal mouse anti-poliovirus antibodies from Thermo Scientific Pierce, specific for the D-antigenic form of type 1, 2, or 3 poliovirus, were diluted 1:1000 in 100 mM carbonate-bicarbonate buffer (pH 9.6) and used to coat 96-well plates overnight at 4 °C. Unbound antibodies were removed with wash buffer (1 × PBS + 0.05% Tween 20), and plates were blocked for 1 h at 37 °C with buffer containing 5% non-fat dry milk in 1 × PBS + 0.05% Tween 20. Blocking buffer was removed, and IPV, diluted to 10 DU/mL in blocking buffer, was added to the wells and incubated at room temperature for 2 h. Unbound IPV was washed from the plates, and serum samples, serially diluted in blocking buffer, was added to the wells and incubated for 2 h at 37 °C. Serum samples were washed from the plate, and polyclonal goat anti-rat IgG HRP-conjugated antibody, diluted 1:3000 in blocking buffer, was added to the wells and incubated for 1 h at 37 °C. Unbound antibody was washed from the plate, and OPD peroxidase substrate was added to the wells and allowed to develop for 30 min at room temperature. The reaction was stopped by addition of 1 M H_2_SO_4_, and absorbance of each well was read at 490 nm using a Tecan multiplate reader. Titer is reported as the lowest dilution that gave an absorbance reading ≥ 2-fold higher than the negative control at the same dilution.

Free IPV injected as two boluses, spaced four weeks apart, was used as the positive control. At each time point, the antigen-specific IgG titers of all four groups were compared using a one-way analysis of variance (ANOVA) with a *post hoc* Dunnett test for multiple comparisons to the positive control.

## 3 Results and discussion

### 3.1 Excipients stabilize liquid IPV formulations over time at physiological temperature

**Fig. 1:**
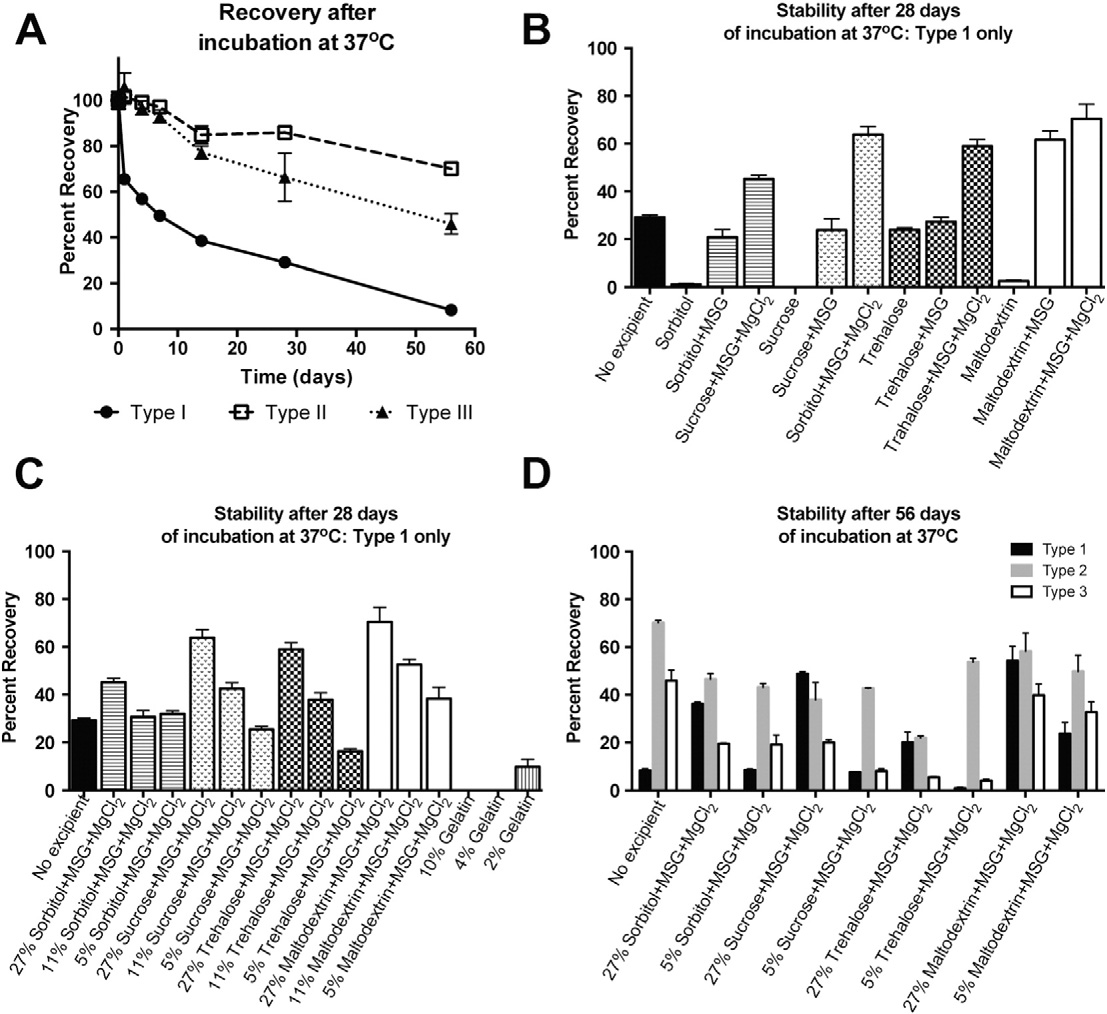
IPV recovery after long-term incubation at 37 °C was improved by excipients. IPV recovery in only buffer was poor after incubation at 37 °C, particularly for serotype 1 (A). However, addition of sugar-based excipients, particularly in combination with MSG and MgCl_2_, greatly improved the 1-month stability of type 1 IPV (B) in a concentration-dependent manner (C). After 56 days of incubation, sucrose- and maltodextrin-based formulations resulted in > 20% and > 35% recovery, respectively, of all three serotypes (D).

Without any additives, IPV rapidly loses D-antigen content when stored at 37 °C (Table 1, Fig. 1A), with 29 ± 1%, 86 ± 3%, and 66 ± 11% recovery of serotypes 1, 2, and 3, respectively, after 1 month of incubation at 37 °C. Various polyols were tested for their ability to stabilize IPV, either by themselves or in combination with monosodium glutamate (MSG) and/or MgCl_2_. Of the four, trehalose was the only one that did not have a negative effect on IPV stability at the concentration tested when used as the sole excipient, with 23.9 ± 0.9%, 79 ± 2%, and 72 ± 4% recovery of types 1, 2, and 3, respectively after 1 month. Most of the sugars gave significantly better results in combination with MSG, and all of them gave the best results after also adding MgCl_2_. It is likely that each component of the combined formulation has a different mechanism of interaction with IPV, resulting in the best thermostability when all three are used together. With a focus on type 1, which was the least stable serotype in initial studies as well as contributing to a high proportion of paralytic poliomyelitis cases [5], sucrose, maltodextrin, and trehalose were the most effective polyols in combination with both MSG and MgCl_2_, with 64 ± 3%, 70 ± 6%, and 59 ± 3% recovery of type 1, respectively, after 1 month at 37 °C (Fig. 1B).

This protective effect was concentration-dependent, as the recovered D-antigenicity decreased with decreasing excipient concentration (Fig. 1C). Although maltodextrin, sucrose, and trehalose all gave similar results at high concentrations and in combination with MSG and MgCl_2_, it is important to note that the thermostabilizing effects of the latter two polyol-based formulations dropped 2.5- to 3.6-fold when their concentration was reduced to 20% of the initially tested formulations. In contrast, the formulation containing maltodextrin, MSG, and MgCl_2_ lost less than half of its efficacy when the concentration was reduced to 20%, resulting in final D-antigen recovery of 38 ± 5%, 79 ± 2%, and 53 ± 3% of types 1, 2, and 3, respectively, after 1 month at 37 °C. After 2 months of incubation at 37 °C with a high concentration of maltodextrin, MSG, and MgCl_2_, IPV D-antigen remained fairly stable, with 54 ± 6%, 58 ± 8%, and 40 ± 5% recovery, respectively (Fig. 1D). Therefore, this formulation was used for further microsphere optimization.

Generally, gelatin did not show stabilizing effects in our hands, with type 1 IPV completely undetectable within 1 month of incubation with 10% gelatin (Fig. 1C). Some D-antigen was recovered when a lower concentration of gelatin was used, suggesting that, under the conditions of this study, gelatin in fact had a destabilizing effect on IPV. It should be noted that many of the polyols alone seemed to have this effect as well and only showed optimal effects in combination with other excipients; however, these excipients could not be tested in combination with gelatin, as the addition of MSG and MgCl_2_ appeared to salt out the protein, resulting in the formation of either a precipitate or a separate phase within the vials. Any trace gelatin left in the IPV_conc_ due to the initial filter-blocking step seemed to have been a negligible amount and caused no measurable difference in IPV recovery (Supplementary Fig. S3).

### 3.2 IPV processing stability studies: water-in-oil emulsion

**Fig. 2:**
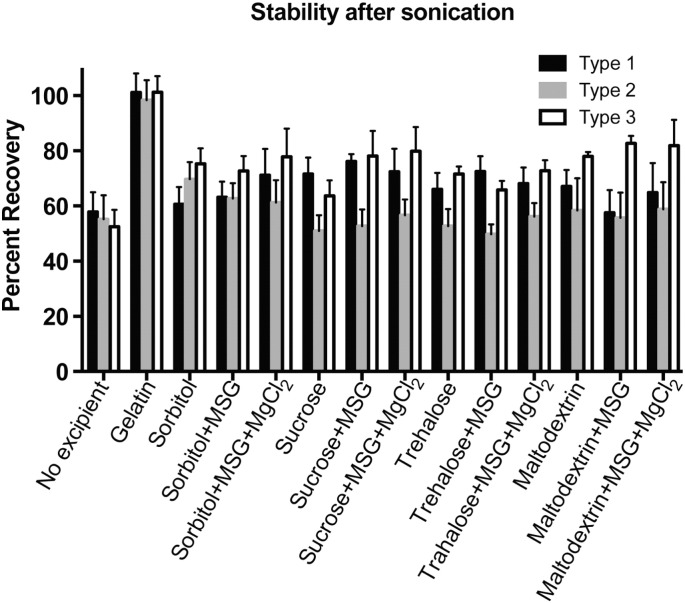
Gelatin protects IPV from damage during sonication. Approximately 40–50% of IPV activity is lost during sonication without addition of excipients. Although small molecule excipients like sugars, salts, and amino acids have some protective effect on stability during this step, gelatin did significantly improve stability when included in the IPV formulation.

For microspheres to be effective, IPV must survive the encapsulation process, including the contact with organic solvents and physical mixing stresses associated with double-emulsion. To test this, IPV was sonicated on ice at low energy for 30 s, then extracted into aqueous buffer to determine the amount of D-antigen that survived the emulsion process. This test was not carried out in the presence of PLGA, because any methods used to extract, degrade, or dissolve the PLGA in order to remove it from the aqueous system also caused denaturation of the IPV. However, while PLGA in the system could affect the sonication process and the IPV recovery, we were still able to compare the relative effects of various excipient formulations on the stability of IPV during sonication. As in previous studies, type 1 IPV had the lowest recovery of the three serotypes (Fig. 2). The inclusion of small polyols, amino acids, or MgCl_2_ salt had no significant effect on IPV stability after emulsification; only gelatin showed a statistically significant (p < 0.01) positive effect on IPV recovery.

It is unsurprising that gelatin had a positive effect on IPV stability during the emulsion process, as one of the major stresses at this step is expected to be the increase in interfacial tension between the oil (organic) and water (aqueous) phases, which can cause denaturation and promote aggregation [26], [27]. Like other proteins, gelatin is amphiphilic in nature and can shield proteins and vaccines from damage at the oil-water interfaces [28], [29]. Sugars, amino acids, and salts did not provide as much protection for IPV during emulsion, although the increase in viscosity of the aqueous phase caused by the addition of such solutes could have contributed to small increases in stability.

### 3.3 IPV processing stability studies: vacuum-drying

Microspheres were dried in the final step of formulation in order to remove excess organic solvents and water and to accurately measure the particle yield. Although lyophilization has been previously used for dried preparations of IPV, many formulations tested in the literature have shown that vacuum-drying, without the initial freezing step of lyophilization, results in better preservation of D-antigenicity [9].

**Fig. 3:**
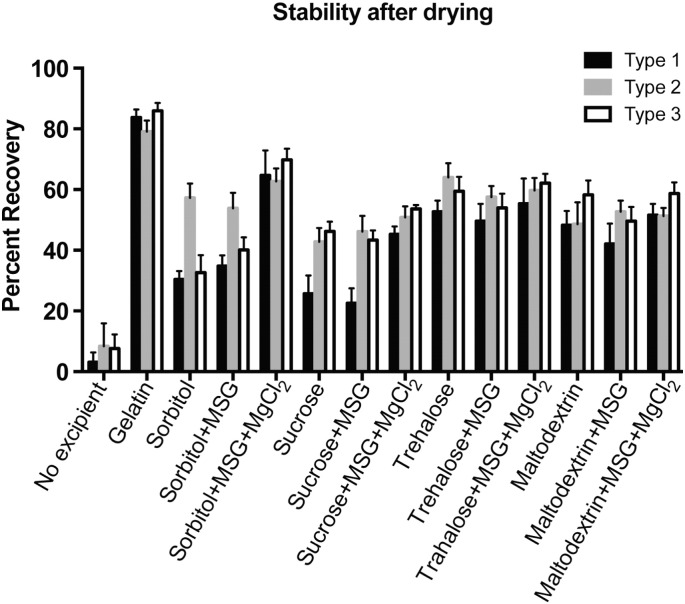
Gelatin and sugar-based excipients protect IPV during dehydration. Without excipients, very little IPV survives being dried under vacuum for 1 h. When mixed first with gelatin, carbohydrates, or a combination of carbohydrates with MSG and MgCl_2_, IPV recovery improves drastically. All experimental groups are statistically significant compared to IPV dried without excipients (< 0.0001).

In our studies, we show that certain excipients or combinations of excipients can drastically improve IPV stability during the drying process. Only 1.0 ± 0.2%, 15 ± 3%, and 1.8 ± 0.3% of D-antigen for type 1, 2, and 3, respectively, was recovered after drying IPV without any additional excipients. All of the excipients tested significantly improved IPV recovery during drying (Fig. 3, p < 0.001 for all compared to no excipient control). Gelatin was again effective in preserving D-antigenicity during drying, in keeping with other work that has found relatively high concentrations of proteins to prevent denaturation during lyophilization or drying by steric hindrance [30]. However, all of the carbohydrate-based excipients also conferred protection, in agreement with results by others showing the ability of sugars to protect proteins, vaccines, or nanoparticles from denaturation during drying [31], [32], [33].

Of the carbohydrates, trehalose and maltodextrin conferred the most protection on their own. When dried with 10% trehalose, 73 ± 6%, 84 ± 5%, and 59 ± 5% of types 1, 2, and 3, respectively, was recovered. When dried with 10% maltodextrin, 88 ± 4%, 99 ± 7%, and 88 ± 5% recovery was observed. However, with the addition of 8.5% MSG and 8.5% MgCl_2_, recovery rates increased for the other sugars as well, with significant improvements in types 1 and 3 recovery for sorbitol- and sucrose-based formulations. It is likely that MgCl_2_ changed the solubility of the proteins in the IPV capsid [34], which resulted here in higher recovery after dehydration. These small changes in relative hydration are expected to have a strong effect, as vacuum-drying is expected to leave some residual water after dehydration [9].

### 3.4 Sugar-based excipient formulations partially stabilize IPV in PLGA microspheres

A number of candidates appeared to be promising excipients for IPV stability, with gelatin yielding the best results for protection during drying and emulsification and sugar-based formulations yielding the best results for IPV incubation over time at 37 °C. In order to evaluate the long-term, controlled release of stable IPV from microspheres, we measured IPV D-antigen activity over time released from PLGA microspheres when co-encapsulated with either gelatin; sucrose, MSG, and MgCl_2_; or maltodextrin, MSG, and MgCl_2_, all of which were top formulations in at least one of the initial stability studies. Representative scanning electron micrographs showed the expected spherical shape and generally smooth morphology for these formulations (Supplementary Fig. S4).

**Fig. 4:**
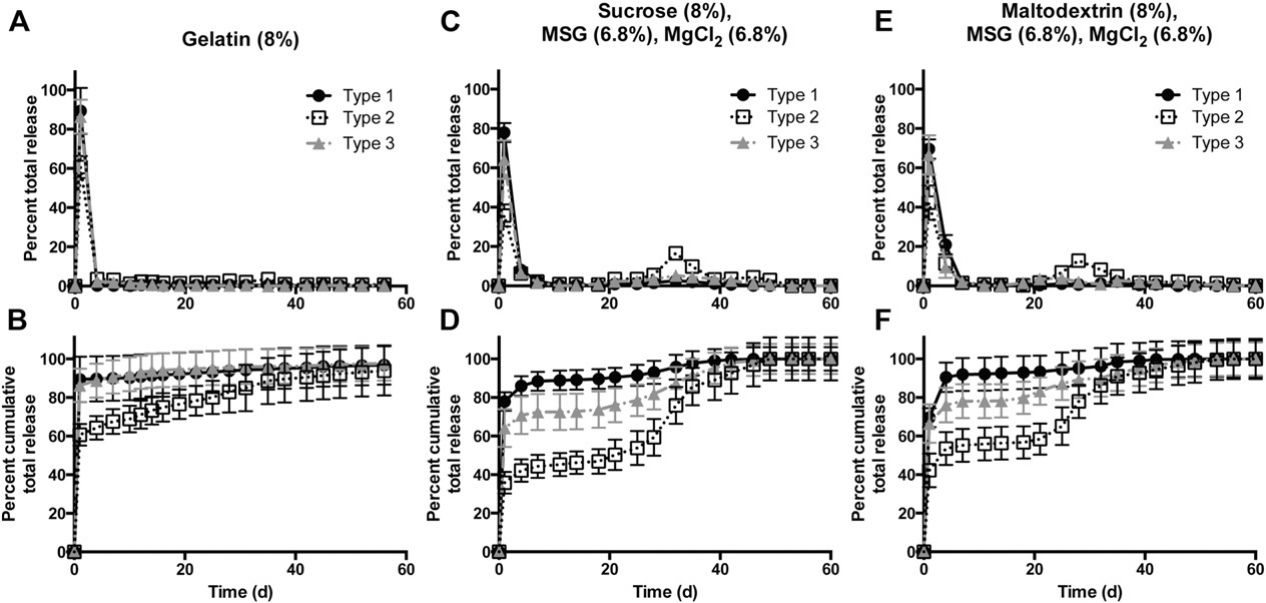
Sugar-based excipients promote long-term IPV release from PLGA microspheres. IPV encapsulated in PLGA microspheres with gelatin (A–B) was released in a strong initial burst. For particles containing sucrose, MSG, and MgCl_2_ (C–D) or maltodextrin, MSG, and MgCl_2_ (E–F), a second burst of type 2 IPV was observed around 25–39 days, with very little of types 1 and 3 released after the initial burst. Graphs in B, D, and F show cumulative IPV release.

An initial burst was measured with all three of the formulations F1, F2, and F3 shown in Fig. 4, which, as expected from the stability studies, showed that IPV could be recovered after the initial steps of microsphere fabrication (emulsification and drying). However, the secondary burst of release, which started after approximately 25 days, was very low for F1 particles containing gelatin as an excipient, with some slight increases seen in serotypes 2 and 3 (Fig. 4A). The secondary burst, starting at 25–28 days, was much more clearly observed from F2 and F3 particles containing either sucrose or maltodextrin along with MSG and MgCl_2_, with this secondary release phase consisting mainly of serotype 2 and only very low levels of serotypes 1 and 3. This multi-phasic release is typical for large macromolecules encapsulated in PLGA-based particles. IPV virions very near the surface of the particles are released quickly in an initial burst, and there is a delay of release while water enters the microsphere matrix and begins to degrade the PLGA by bulk erosion. Once enough of the PLGA has degraded for channels to form through the microsphere matrix, release accelerates again in a second burst.

Gelatin-containing particles showed a large initial burst but no significant release thereafter, with nearly 100% of all release occurring in the first few days (Fig. 4A-B). This was expected and validates use of the liquid incubation assay used to measure stability: gelatin alone as an excipient improved IPV recovery after emulsification and drying but showed very little ability to stabilize IPV in an aqueous, 37 °C environment over time. Although gelatin has been found in some systems to stabilize certain antigens against stresses of heat and moisture [35], other studies have reported findings similar to ours, with gelatin and viscosity-increasing agents protecting proteins from interfacial tension [27] but having a very low or even a negative effect on protein stability [36], [37]. Both sucrose and maltodextrin in combination with MSG and MgCl_2_ are superior for long-term IPV stability inside PLGA particles in an aqueous 37 °C buffer, confirming the long-term thermostability results seen independently of PLGA particles. As a result, combinations of sucrose or maltodextrin with MSG and MgCl_2_ were pursued for further study. However, even with the carbohydrate-based excipients, only type 2 IPV was detected in the second burst in high amounts (Fig. 4C–F), and very little of types 1 and 3 were released after the first four days.

Formulations F1, F2, and F3 all showed similar encapsulation efficiencies (Table 3), with 77–84% encapsulation of each serotype in the F1 gelatin-containing particles and 64–69% encapsulation in the particles containing sugar-based excipients. Because of the low IPV mass concentration and the high excipient:IPV mass ratios used in these microspheres, non-specific protein assays and chromatographic methods could not be used to quantify protein release, and an ELISA specific for denatured IPV was used instead, and the small differences in the measured IPV encapsulation in F1 particles could be due in part to the minor effect of the excipient mixtures on the binding equilibrium between IPV and ELISA antibodies. It is likely, therefore, that the differences in release seen in F1 particles compared to F2 and F3 are due primarily to long-term stabilization capabilities of the excipient mixtures. Moreover, for each formulation, all three serotypes were encapsulated to a similar extent, which is expected from the physical and chemical similarity of the three types of virions. Thus, the very low amount of delayed release of types 1 and 3 suggests that IPV stability in PLGA microspheres during the release study was affected by other factors that were not present in the earlier thermostability studies, which showed up to 70–72% recovery of types 1 and 3 after 1 month. Therefore, stresses specific to the PLGA microenvironment were explored.

### 3.5 IPV pH-sensitivity studies

Because PLGA degrades into lactic acid and glycolic acid, the pH around and within the microspheres decreases dramatically over time in an aqueous environment, with some studies finding that the internal pH of PLGA-based microspheres can reach as low as 1.5 [38]. Because IPV is trapped inside the particles for weeks to months before being released, it was necessary to assess its sensitivity to the pH extremes that it would experience over the relevant time period. Initial studies used fluorescently labeled IPV (AF680-IPV) encapsulated in PLGA502H microspheres with 10 μL of 20% sucrose, 17% MSG, and 17% MgCl2 as the excipients, and IPV release was measured by fluorescence instead of by an antibody-based assay, thereby allowing total IPV release kinetics to be assessed without taking into account IPV stability over time.

**Fig. 5:**
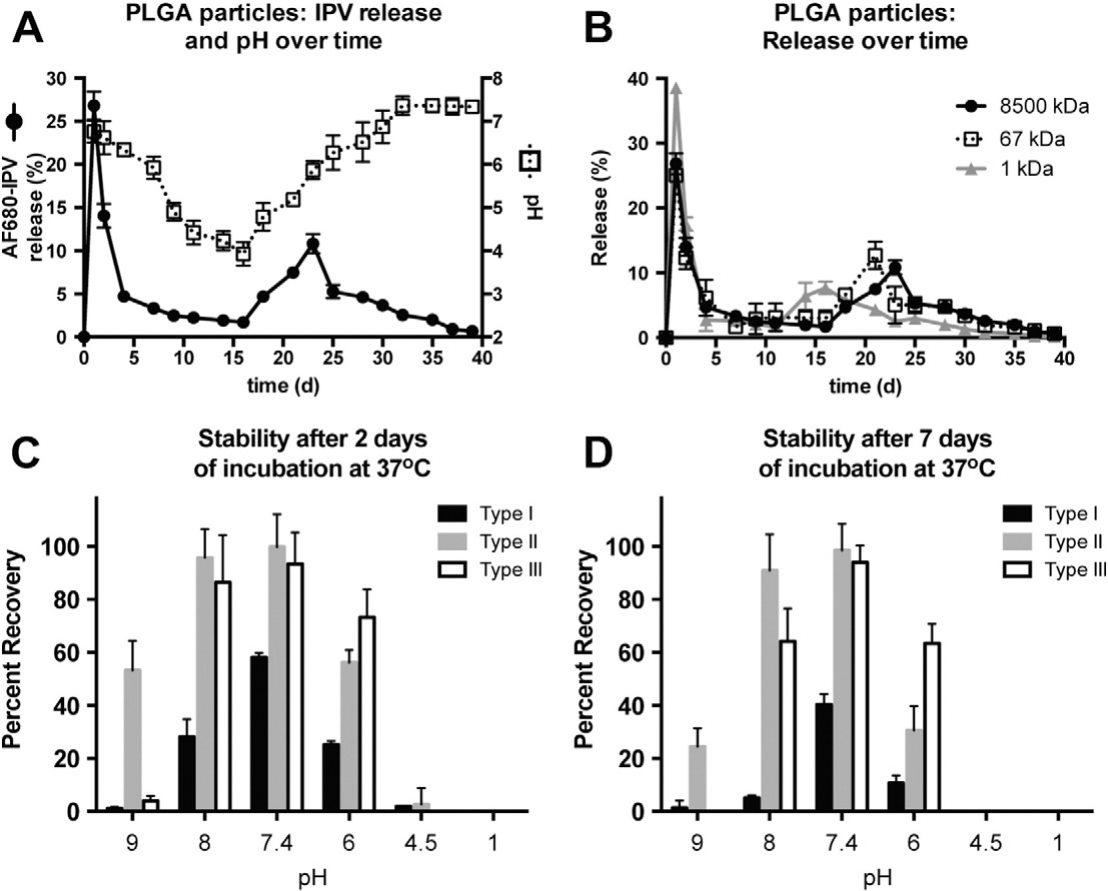
Low pH in degrading PLGA microspheres decreases IPV stability over time. The pH surrounding PLGA microspheres decreases over the course of a release study, with the lowest pH measured just before IPV is released in measurable amounts (A). The difference in release kinetics between cargo of varying molecular weight from approximately 1 to 8500 kDa suggests that IPV would be trapped inside the degrading particles before acidic protons are released and would therefore experience a low-pH environment (B). Stability studies show that IPV, particularly type 1, is very unstable in even slightly basic or acidic pH after 2 days (C) or 7 days (D) of incubation at 37 °C, further emphasizing the need to relieve the acidity within particles.

As seen in Fig. 5A, the pH of the release medium at each time point steadily decreased over the course of the release study, reaching < 4 after 16 days of release, indicating that PLGA is degrading and acidifying the release buffer over this period. Following this, the pH slowly rises again and finally stabilizes at 7.0–7.4 after 30 days. It is important to note that the pH reported is that of the release medium, *i.e.* the pH outside the particles. In contrast, the internal pH of microspheres can be affected by various factors, including the degree to which the particles swell with water, buffering salts, and the rate of diffusion of water, excipients, and protons into and out of the particles. The external pH can critically affect the stability of IPV that has been released and remains in the release medium for hours or days until a time point is taken; however, it does not precisely reflect the internal microsphere pH, which could be particularly important to the stability of IPV that is yet unreleased. The continual decrease in pH of the release medium over the first 2–3 weeks followed by its steady rise seems to indicate that acidic protons can diffuse out of the particles, but it is reasonable to assume that the proton concentration within the particles in the first weeks is equal to or higher than that in the outside medium. Although the buildup of internal acidity contributes to the characteristic, pulsatile pattern of release from PLGA particles, it could also contribute to IPV instability at later time points.

When AF680-IPV is encapsulated, a large portion is released within the first few days (41% within 2 days), followed by little or no release and then a second burst of release between 18 and 35 days. Importantly, a large amount of the IPV has not yet been released from the microsphere when the pH is measured to be at its lowest in the external environment, implying that the internal pH near the unreleased IPV is likely to be very low as well.

It is possible that the IPV is released in a later second burst than the acidic protons because of the difference in size between the two. Each IPV virion has a molecular mass of approximately 8500 kDa [39]. It is reasonable that a much larger macromolecule requires more of the particle to have degraded, forming larger channels through the polymer matrix, in order to be able to diffuse into the release medium. This hypothesis was tested by comparing the release kinetics of AF680-IPV, AF680-BSA, and AF680 free dye, with approximate molecular weights of 8500 kDa, 67 kDa, and 1 kDa, respectively (Fig. 5B). The shift in the second burst of release is clearly related to the size of the molecule being encapsulated, with smaller molecules being released earlier. Since IPV releases later than the small molecules, it is likely to be trapped in the acidic internal environment for long periods of time before it can diffuse into the release medium.

Whether IPV is released or still trapped in the PLGA microsphere matrix, the surrounding pH can have an important effect on its stability (Fig. 5C–D). At low concentrations, as IPV would be after release into the buffer, type 1 IPV loses stability rapidly even at neutral (7.4) pH, with only 58 ± 1% remaining after 2 days at 37 °C and 40 ± 2% after 7 days. Even small deviations from neutral pH cause type 1 IPV to denature even more quickly, with 5 ± 1% and 11 ± 2% recovery at 37 °C after 7 days at pH 8 and 6, respectively. D-antigen was < 5% for all serotypes after incubation for 2 or more days at pH 4.5. As seen in the earlier experiments, types 2 and 3 both tended to be more stable than type 1, with no statistically significant loss in D-antigen content after 7 days of incubation at pH 7.4. However, type 2 stability decreased upon incubation in an acidic medium, with only 31 ± 5% recovery after 7 days at pH 6. While type 3 appeared to be slightly less sensitive to acid than types 1 and 2 (63 ± 4% recovery after 7 days at pH 6), lost stability when kept in even slightly basic pH (64 ± 7% recovery after 7 days at pH 8). The sensitivity of IPV to basic as well as acidic environments is important for understanding how PLGA degradation affects the IPV cargo and how best to compensate for acid buildup using basic excipients.

### 3.6 Basic small molecules reduce IPV damage over time due to PLGA degradation products

In order to combat IPV loss due to increasing acidity from the degrading PLGA, basic excipients were added to the microsphere formulations. Arginine, a basic amino acid that has been found to stabilize viruses and vaccines [40], [41], was dissolved in the same aqueous phase as the IPV and excipients. Mg(OH)_2_, a basic and poorly soluble salt [42], [43], can buffer the acidic environment and was dispersed through the first PLGA-containing oil phase by sonication. Neither of these additives showed a strong effect on encapsulation efficiency (Table 3).

**Fig. 6:**
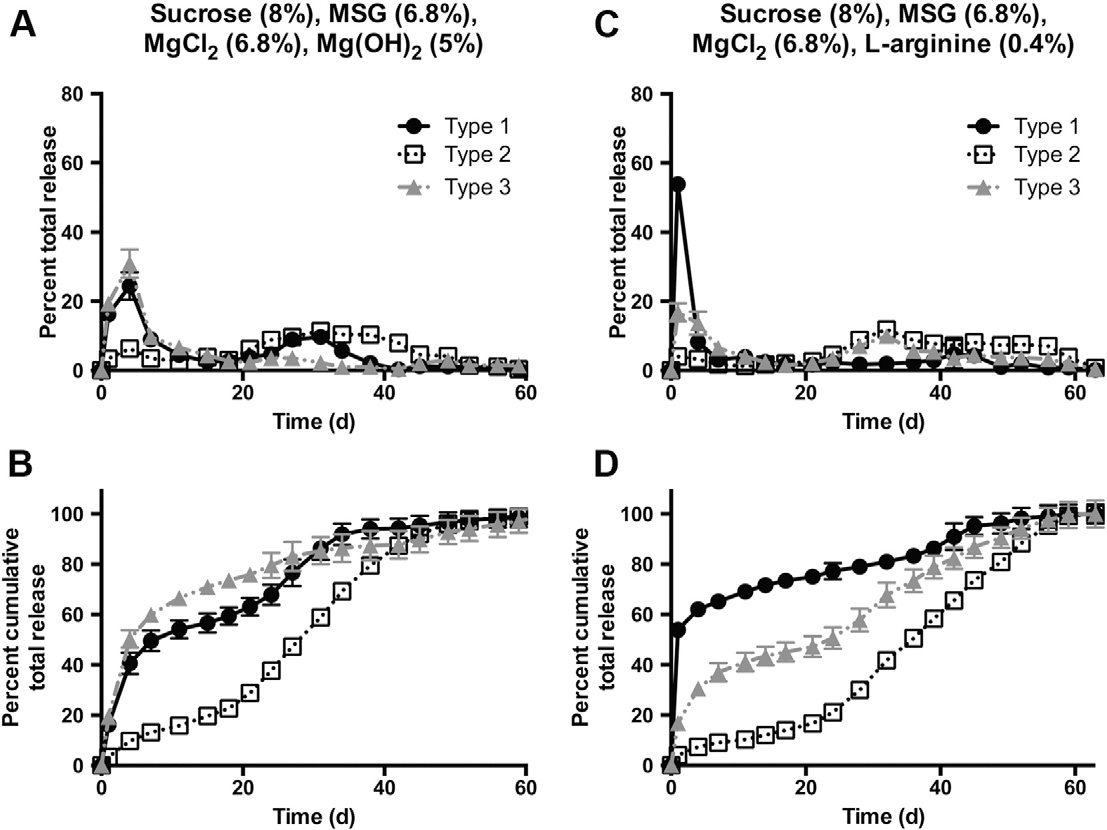
Co-encapsulation of basic excipients improves IPV stability at late time points. Adding Mg(OH)_2_ (A–B) or arginine (C–D) improved type 1 and 3 stability compared to particles without basic excipients. However, neither type 1 nor type 3 was able to approach the high release seen with type 2. Cumulative release normalized to total release is shown in B and D.

Some type 1 or type 3 release was seen in the secondary burst of Mg(OH)_2_- and arginine-containing particles (Fig. 6), formulations F4 and F5, respectively. In the case of both particles, the peaks corresponding to both the initial and secondary bursts are broader, resulting in less narrow pulses of release. Because the characteristic pulsatile release from PLGA particles is due in part to the accelerated degradation of the ester bond as acid accumulates within these particles, it is unsurprising that molecules that reduce accumulation of acid would also result in more continuous release. It is significant that high amounts of type 2 IPV still remained stable and in D-antigen form as long as 40–50 days after the start of the release study.

From Mg(OH)_2_-containing particles, type 1 release in the second burst was 4.4-fold higher than from particles with only sucrose, MSG, and MgCl_2_; type 2 also had a 6.4-fold improvement in the second burst, and type 3 had only 0.6-fold as much release in the second burst (Table 4). From arginine-containing particles, type 1 release in the second burst was not improved, but types 2 and 3 saw 5.1- and 2.3-fold greater release, respectively, in the second burst. Moreover, type 1 IPV shows very little release at all, initially or at later time points, from arginine-containing particles. Because arginine is basic and water-soluble, it is possible that it raises the pH of the aqueous phase too far above neutral, causing denaturation of the more sensitive IPV serotypes. In addition, the type 1 and 3 secondary bursts, although higher than in previous formulations, are still quite small, and only type 2 was released in amounts high enough for a human therapeutic dose (Table 4) equivalent to two standard clinical boluses. Therefore, while F4 and F5 may not be ideal for stabilization and extended release of all three IPV serotypes, these formulations could be highly effective for stabilization of type 2 IPV if monovalent IPV were to be encapsulated.

### 3.7 Addition of cationic Eudragit E allows pulsatile, controlled release of IPV

To address the hypothesis that accumulation of acid inside the particles was still one of the major problems for achieving late release of IPV, Eudragit E PO (EPO) was doped into the polymer-containing first oil phase. Because of the amines in its backbone, EPO can act as a base and help to buffer the internal environment. However, unlike small soluble molecules such as arginine, EPO is poorly water soluble at neutral or slightly acidic pH, which prevents it from causing denaturation of IPV by making the aqueous compartment excessively basic; at pH < 3, EPO becomes soluble in aqueous solutions at 20 mg/mL or lower concentrations. Therefore, when it is blended with PLGA and incorporated into the microspheres, EPO does not initially cause major changes in pH, as it is incorporated into the aprotic organic phase during fabrication. However, upon hydration of the microspheres during *in vitro* release studies or after *in vivo* injection, EPO causes an increase in the local pH of the PLGA-containing compartment, which accelerates local PLGA degradation by base-catalyzed hydrolysis. As PLGA is hydrolyzed, EPO becomes progressively more protonated as the environment increases in acidity, and when acidic protons accumulate to a critical level, EPO dissolves into the aqueous phase and can then diffuse out of the microsphere. Because EPO forms part of the microsphere matrix, its dissolution creates channels large enough through which protons as well as the cargo of interest, IPV, can diffuse out, rather than being confined within the acidified particle.

**Fig. 7:**
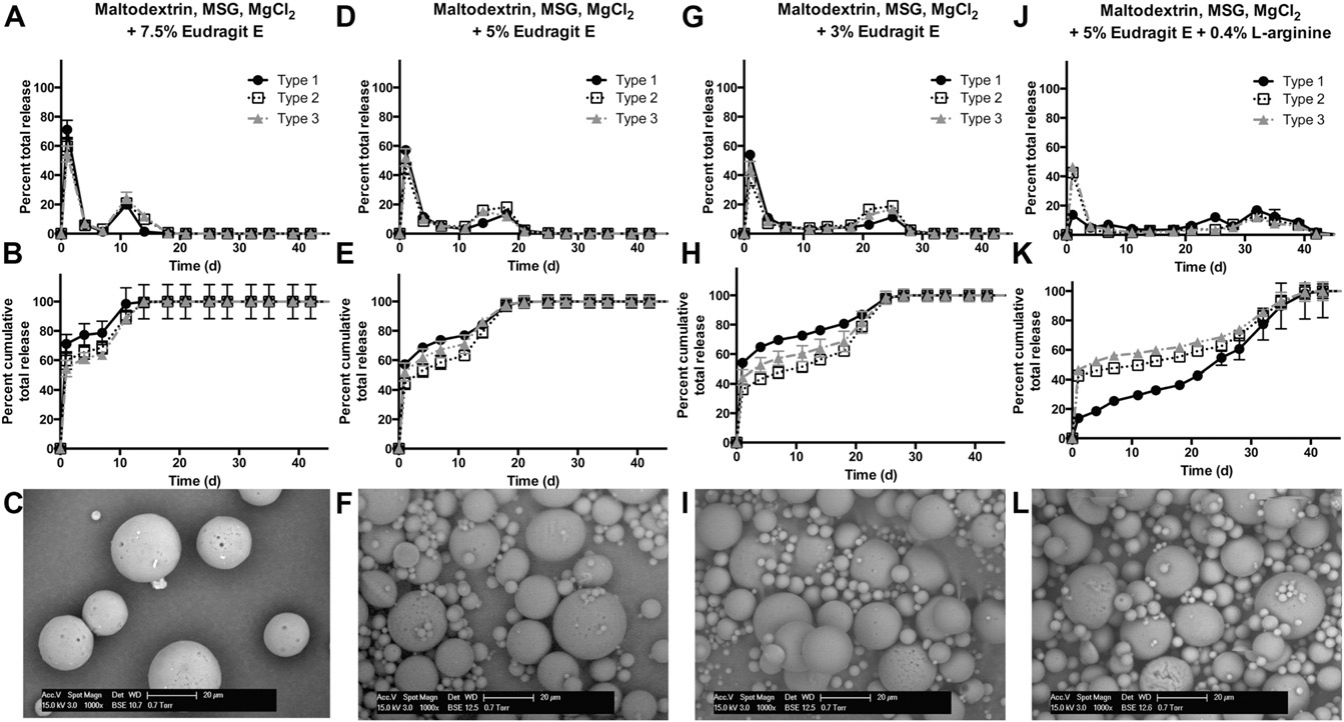
Eudragit E-doped particles show release of stable IPV at 2–3 weeks. By incorporating the basic, acid-soluble EPO into the PLGA-based particles, IPV could be protected from accumulating acid and be released in stable form. Decreasing the EPO concentration from 7.5% (A–C) to 5% (D–F) to 3% (G–I) allowed the kinetics of the second burst to be tuned. Adjusting the pH by adding arginine (J–L) also slowed the second burst of release.

We found that doping in EPO significantly improved the release of stable IPV, particularly types 1 and 3, after several days or even weeks (Fig. 7). For instance, formulation F7, containing maltodextrin, MSG, MgCl_2_, and 5% EPO (Fig. 7D-F), shows improvements of 7.8-, 0.9-, and 3.2-fold in type 1, 2, and 3 release, respectively, in the second burst. The release profile with this method is much more pulsatile than many of the profiles achieved before, with the second burst largely taking place over a span of approximately 1 week or less (Fig. 7). This is likely because the inclusion of EPO accelerates degradation of the PLGA, resulting in complete or near-complete IPV release within a short period of time. All EPO-containing formulations F6–F9 had similar encapsulation efficiencies ranging from 60 to 83% for each serotype (Table 3). Particularly in the cases of F7 and F8, with 5% and 3% EPO, respectively, the amount of D-antigen recovered from the relatively unstable types 1 and 3 over the course of 1 month of release was much higher than in previously tested formulations.

Interestingly, using EPO as a dopant allows the release kinetics to be tuned easily by simply altering the ratio of EPO to PLGA. With 7.5% EPO added to the formulation, the secondary burst peaks at approximately 11 days, while 5% and 3% EPO result in a second peak after 14–18 days and 25 days, respectively. Addition of a basic excipient, arginine, to the formulation delays the acidification and therefore the EPO dissolution in the surrounding aqueous solution, resulting in a second peak after 32 days. The ability to tune kinetics greatly increases the utility of this technology, as it allows the antigen to be released at desired time points over the course of a month.

**Fig. 8:**
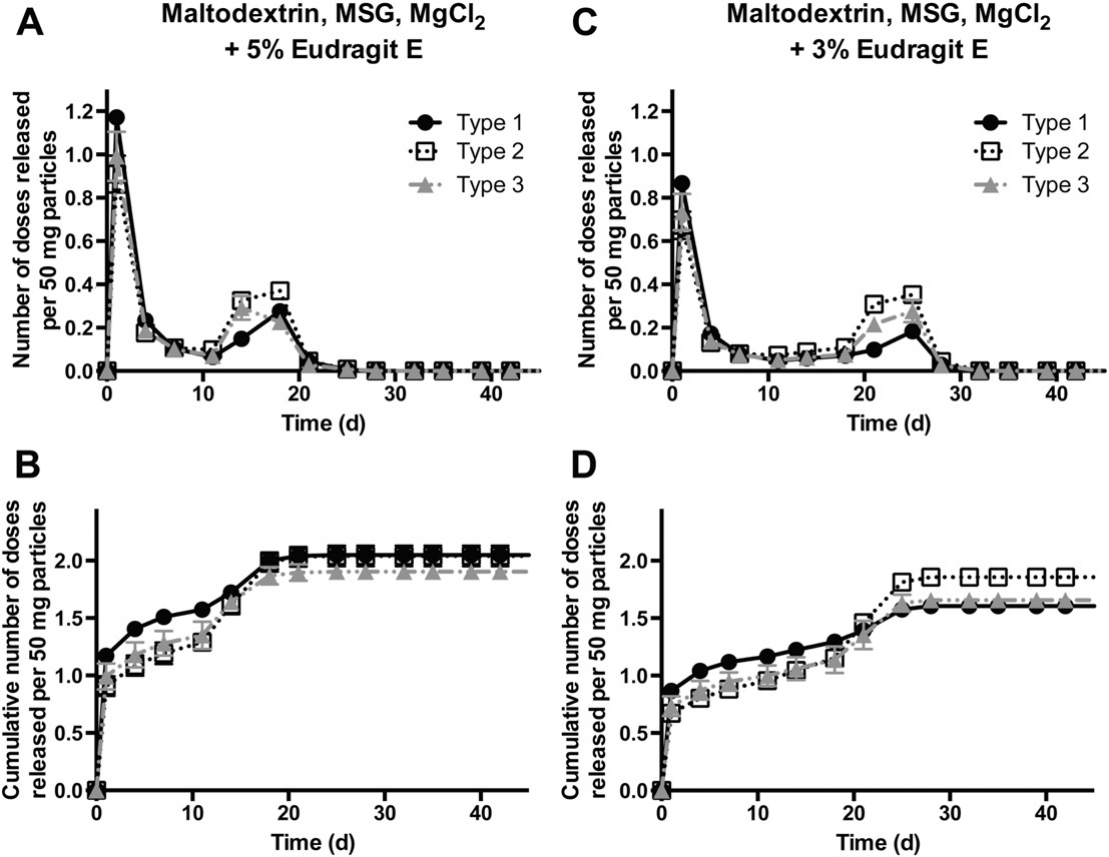
Formulations F7 and F8 release therapeutic doses of IPV in two distinct bursts. Both of the leading EPO-containing formulations release the equivalent amount of IPV as a standard dosing regimen consisting of two bolus injections. A and C show the number of human doses released by an injectable mass of particles, with one human dose defined as a single bolus; B and D show the cumulative number of doses released.

Importantly, the amount of IPV released by the EPO-containing formulations in both the initial and secondary bursts approaches or exceeds the amount of IPV normally delivered in two bolus doses over 1–2 months by current clinical standards (Fig. 8). For example, from 50 mg of F7, with maltodextrin, MSG, MgCl_2_ and 5% EPO as excipients, 1.4, 1.1, and 1.2 human doses of types 1, 2, and 3, respectively, were released in an initial burst, with 0.5, 0.8, and 0.6 doses released in a separate second burst. This indicates that these particles are able to release a therapeutically relevant amount of vaccine within the 1–2 month timeframe that is currently recommended by the WHO. These results are very promising, as the amount of injected particles can be easily adjusted so that the formulations release exactly one human dose per time point.

### 3.8 Efficacy of IPV-containing microspheres *in vivo*

While *in vitro* studies showed that microspheres such as formulations F7 and F8 could release pulses of IPV over one month, the ability to elicit an immune response *in vivo* is critical to their potential use as a clinical vaccine. To show their efficacy in a rodent model, rats were injected IM with boluses of IPV, mimicking the current clinical administration method, or with IPV-containing microsphere formulation F8.

**Fig. 9:**
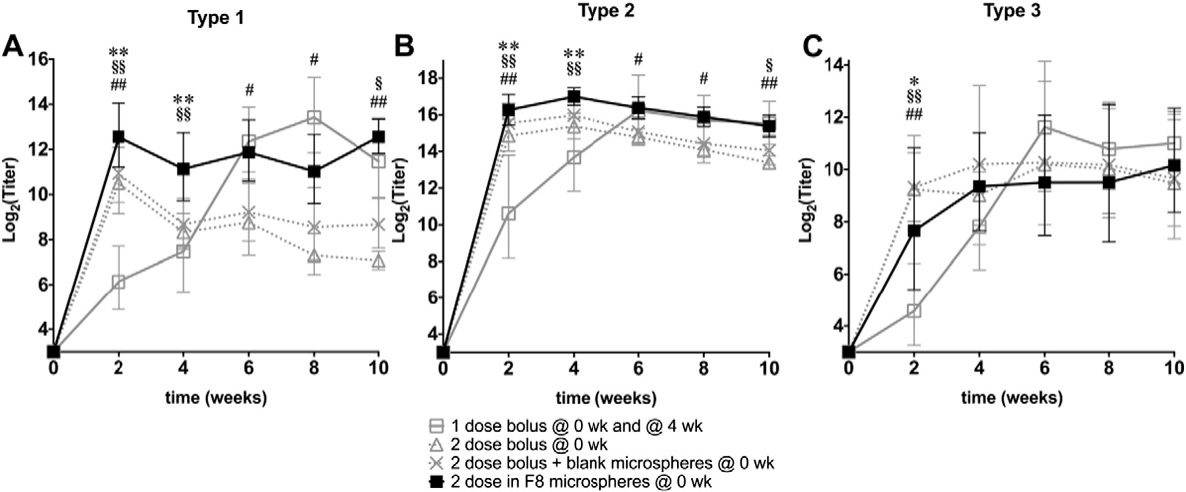
Formulation F8 elicits non-inferior immune response compared to bolus injections of IPV. Humoral immune response elicited by a single bolus of IPV (Δ open triangle), a single bolus of IPV co-injected with empty particles (✕ cross), two boluses of IPV (☐ open square), or IPV encapsulated in F8 microspheres (■ solid square) was measured in rats. Antibody titers specific for type 1 (A), type 2 (B), or type 3 (C) poliovirus are plotted on a log-2 scale as geometric mean ± 95% confidence interval. Statistically significant differences from the positive control (two boluses of IPV) are marked as: * < 0.05 and ** < 0.01 (IPV encapsulated in F8 microspheres); § < 0.05 and §§ < 0.01 (single bolus co-injected with empty particles); and # < 0.05 and ## < 0.01 (single bolus).

As expected, injecting a single bolus containing two rat doses elicited binding antibodies to all three serotypes (Fig. 9). However, when the boluses are spread over time, with one rat dose administered initially (t = 0) and then again 4 weeks later, the resulting antibody response equals or exceeds that of a single bolus, highlighting the importance of a second antigen presentation for vaccine efficacy. Interestingly, F8 microspheres elicited a strong antibody response against type 1 IPV and, even at early time points, before all of its cargo is expected to have been released, surpassed the humoral response seen from the single bolus containing the full two rat doses. While this improvement over the standard bolus is not seen to a statistically significant extent in the type 2 and type 3 responses, it is interesting to note that the antibody titers initially elicited by a single bolus are similar to those elicited by the F8 microsphere formulation, which contains the same total IPV dosage but has not yet released all of its cargo.

This apparent adjuvant effect of the microspheres has been well-documented in other systems [44], [45], [46] and may play a role in the effectiveness of the current formulations studied here for IPV delivery. Although several mechanisms may contribute to this effect, co-injection of a bolus with blank microspheres showed only minor differences compared to the bolus alone (Fig. 8), suggesting that the adjuvancy is not caused simply by the presence of the particles themselves or any non-specific reaction to the particles or their degradation products. No clear signs of toxicity were observed after injection with microspheres. As others have found that the kinetics of antigen presentation can have an important effect on the immune response [47], [48], it is likely that the particular release kinetics of IPV from microspheres contribute to the induction of a stronger antibody response compared with a single bolus.

The boluses administered 4 weeks apart do show a strong increase in antibody titers after the second dose. For types 1 and 2, boluses administered 4 weeks apart elicited a significantly stronger immune response than a single bolus alone at all time points. However, the titers elicited even by two boluses again fall more quickly than those elicited by the microspheres, and 10 weeks after the initial injection, antibodies against the IPV boluses were statistically equivalent to those against F8 for all three serotypes. Interestingly, despite the pulsatile release profile of F8 microspheres seen *in vitro*, *in vivo* they elicited high antibody titers within a few weeks and then maintained this high level for several weeks, in contrast to the distinct inflection at the time of the booster that is seen in the group immunized with two separate boluses. This may be a consequence of the broader peaks of IPV release by F8 microspheres compared to the very brief IPV presentation in bolus groups. Most importantly, however, this broad release may contribute to a positive effect on the immune response.

## 4 Conclusions

Single-injection vaccines are of high interest in the global health field to improve compliance and aid in eradication efforts. IPV, which possesses poor thermostability on its own and in current clinical formulations, was shown here to have markedly higher stability at 37 °C upon incubation with excipients containing a carbohydrate, MSG, and MgCl_2_, with increased recoveries of 2.4-fold after 1 month for type 1 IPV. With the aid of excipients, IPV could be encapsulated in PLGA-based microspheres and released *in vitro* in two bursts separated by approximately 1 month. In addition to thermostabilizing excipients, basic, cationic dopant Eudragit E polymer caused clinically relevant amounts of stable IPV release upon degradation of the PLGA matrix, thus minimizing the number of required vaccine administrations. Critically, *in vivo* studies showed that this technology can induce a potent immune response in animal models by measurement of IPV-specific binding antibodies, with a quick induction and long duration of high antibody titers. With a single injection, the top microsphere formulation showed non-inferior antibody response compared to two separate bolus injections after 10 weeks. An ideal vaccine product using this platform could potentially be stored in dry form, reconstituted before use, and injected into a patient, requiring only a single visit from a healthcare professional for long-lasting protective immunity. Therefore, this technology can potentially serve as an important tool to aid in the eradiation of polio and other infectious diseases for the improvement of global health.

## Figures and Tables

**Table 1:** Stabilizing excipients tested for IPV thermostability studies.

Sugars/sugar alcohols	Amino acids	Proteins	Divalent salt	Mean IPV D-antigen recovery after		
1 month at 37 °C (%)		
Type 1	Type 2	Type 3
–	–	–	–	29%	86%	66%
10% sorbitol	–	–	–	1%	0%	31%
10% sorbitol	8.5% MSG	–	–	21%	86%	31%
10% sorbitol	8.5% MSG	–	8.5% MgCl_2_	45%	83%	38%
4% sorbitol	3.4% MSG	–	3.4% MgCl_2_	31%	77%	50%
2% sorbitol	1.7% MSG	–	1.7% MgCl_2_	32%	73%	52%
10% sucrose	–	–	–	0%	0%	16%
10% sucrose	8.5% MSG	–	–	24%	79%	27%
10% sucrose	8.5% MSG	–	8.5% MgCl_2_	64%	78%	37%
4% sucrose	3.4% MSG	–	3.4% MgCl_2_	42%	69%	54%
2% sucrose	1.7% MSG	–	1.7% MgCl_2_	25%	68%	54%
10% trehalose	–	–	–	24%	79%	72%
10% trehalose	8.5% MSG	–	–	27%	56%	59%
10% trehalose	8.5% MSG	–	8.5% MgCl_2_	59%	51%	52%
4% trehalose	3.4% MSG	–	3.4% MgCl_2_	38%	80%	71%
2% trehalose	1.7% MSG	–	1.7% MgCl_2_	16%	78%	32%
10% maltodextrin	–	–	–	3%	33%	2%
10% maltodextrin	8.5% MSG	–	–	62%	80%	55%
10% maltodextrin	8.5% MSG	–	8.5% MgCl_2_	70%	83%	65%
4% maltodextrin	3.4% MSG	–	3.4% MgCl_2_	53%	76%	61%
2% maltodextrin	1.7% MSG	–	1.7% MgCl_2_	38%	79%	53%
–	–	10% gelatin	–	0%	49%	18%
–	–	4% gelatin	–	0%	54%	32%
–	–	2% gelatin	–	10%	64%	41%

**Table 2:** Excipients co-encapsulated with IPV in PLGA microspheres. Percentages refer to the mass ratio of excipients to the PLGA microspheres.

Formulation	Number-weighted mean diameter (μm)	Sugars/sugar alcohols	Amino acids	Proteins	Divalent salt	Other
F1	5.8 ± 3.6	–	–	8% gelatin	–	–
F2	10.7 ± 3.4	8% sucrose	6.8% MSG	–	6.8% MgCl_2_	–
F3	10.2 ± 3.3	8% maltodextrin	6.8% MSG	–	6.8% MgCl_2_	–
F4	10.3 ± 3.4	8% sucrose	6.8% MSG	–	6.8% MgCl_2_	5% Mg(OH)_2_
F5	8.7 ± 3.5	8% sucrose	6.8% MSG	–	6.8% MgCl_2_	0.4% l-arginine
F6	8.3 ± 3.5	8% maltodextrin	6.8% MSG	–	6.8% MgCl_2_	7.5% EPO
F7	7.7 ± 3.4	8% maltodextrin	6.8% MSG	–	6.8% MgCl_2_	5% EPO
F8	11.2 ± 3.4	8% maltodextrin	6.8% MSG	–	6.8% MgCl_2_	3% EPO
F9	10.5 ± 3.3	8% maltodextrin	6.8% MSG	–	6.8% MgCl_2_	5% EPO, 0.4% arginine

**Table 3:** IPV encapsulation efficiency in microspheres. Percent encapsulation efficiency is normalized to theoretical initial loading. Percent recovery of D-antigen during release is normalized to the actual measured loading. Data are represented as mean ± standard deviation (n = 3).

Formulation	IPV encapsulation efficiency (%)			Total IPV encapsulated (number of doses/50 mg)			D-antigen recovery during release (%)		
	Type 1	Type 2	Type 3	Type 1	Type 2	Type 3	Type 1	Type 2	Type 3
F1	84 ± 4	81 ± 5	77 ± 2	27 ± 1	26 ± 2	25 ± 1	2.3 ± 0.3	4.6 ± 0.4	4.1 ± 0.3
F2	69 ± 4	67 ± 4	67 ± 5	22 ± 1	21 ± 1	21 ± 2	2.7 ± 0.1	5.6 ± 0.6	4.2 ± 0.2
F3	68 ± 7	69 ± 11	64 ± 6	22 ± 2	22 ± 3	20 ± 2	4.6 ± 0.2	9.1 ± 0.4	4.9 ± 0.3
F4	66 ± 5	63 ± 6	70 ± 6	21 ± 1	20 ± 2	22 ± 2	2.4 ± 0.3	23 ± 1.2	2.2 ± 0.2
F5	74 ± 4	75 ± 4	69 ± 5	24 ± 1	24 ± 1	22 ± 2	0.4 ± 0.2	14 ± 0.5	4.1 ± 0.5
F6	82 ± 12	83 ± 9	83 ± 2	26 ± 4	27 ± 3	27 ± 1	4.6 ± 0.9	7.5 ± 0.9	4.5 ± 0.3
F7	70 ± 8	71 ± 4	72 ± 7	22 ± 2	23 ± 1	23 ± 2	9.0 ± 0.4	8.8 ± 0.7	8.3 ± 0.7
F8	75 ± 6	73 ± 3	70 ± 2	24 ± 2	23 ± 1	23 ± 1	6.7 ± 0.4	8.2 ± 1.2	7.5 ± 0.9
F9	61 ± 6	60 ± 5	60 ± 10	20 ± 2	19 ± 2	19 ± 3	1.1 ± 1.2	24 ± 2.3	8.3 ± 0.9

**Table 4:** Percent of total IPV release measured during the first and second bursts.

Formulation	Initial burst (%)			Second burst (%)			Number of human doses released in total by 50 mg particles		
Type 1	Type 2	Type 3	Type 1	Type 2	Type 3	Type 1	Type 2	Type 3
F1	90	64	88	3	14	2	0.6	1.2	1.0
F2	86	42	71	8	46	22	0.6	1.2	0.9
F3	90	53	76	6	42	17	1.0	2.0	1.0
F4	49	13	60	40	77	25	0.5	4.6	0.5
F5	67	9	34	27	83	53	0.1	3.4	0.9
F6	77	66	61	22	31	36	1.2	2.0	1.2
F7	69	53	62	23	37	29	2.0	2.0	1.9
F8	65	43	52	20	54	18	1.6	1.9	1.7
F9	20	46	53	70	50	34	0.2	4.6	1.6

## Appendix A Supplementary data

Supplementary material Image 2
