# Supplementary material for: Thermostabilization of inactivated polio vaccine in PLGA-based microspheres for pulsatile release
Source: J Control Release. 2016 Jul 10;233:101–13. doi: 10.1016/j.jconrel.2016.05.012 (PMC4925381; doi:10.1016/j.jconrel.2016.05.012)
Supplement: Supplementary file 1 — Supplementary material [file mmc1.pdf]

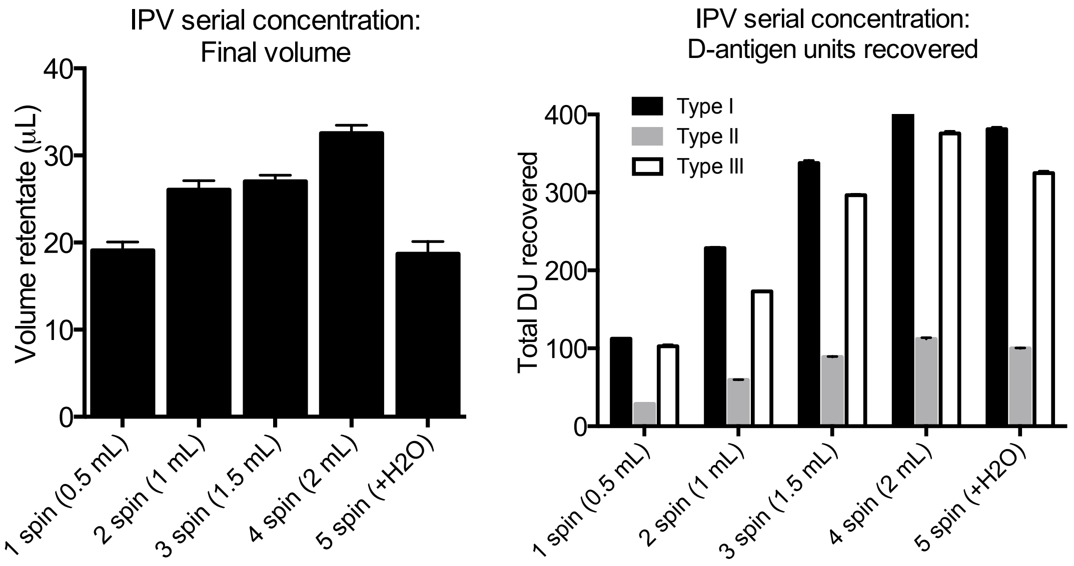

Supplementary Figure S1. tIPV was concentrated by centrifugal filtration to form IPV_{conc}, finally consisting of 19±1 μL of IPV at 20060 DU/mL, 5260 DU/mL, and 17089 DU/mL of type 1, 2, and 3. Each centrifugation step is represented on the x-axes, with 0.5 mL tIPV stock added in each step and the final filtration using water to remove excess salts and other small molecules from the solution.

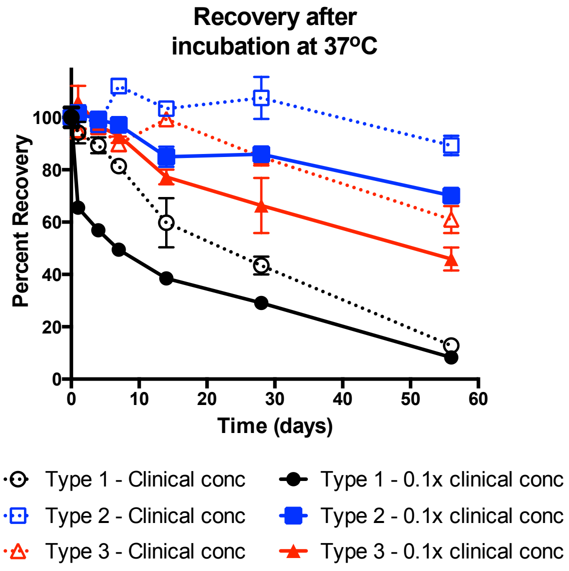

Supplementary Figure S2. IPV was incubated in 1xPBS at 37°C for 2 months and tested for stability over time via ELISA. Because stability was harder to maintain at low concentrations, this low concentration (0.1x of the normal clinical concentration) was used for thermostability studies with excipient formulations.

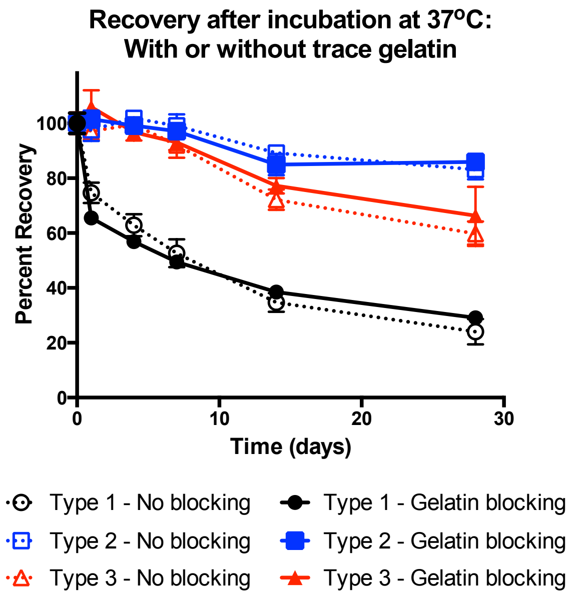

Supplementary Figure S3. Because the filters used for concentration are blocked with gelatin to prevent non-specific adsorption to the filter, trace amounts of gelatin may remain in the final product. Any remaining gelatin has no statistically significant effect on IPV thermostability, and gelatin was therefore continued to be used for blocking filters in order to increase the IPV D-antigen recovery after concentration.

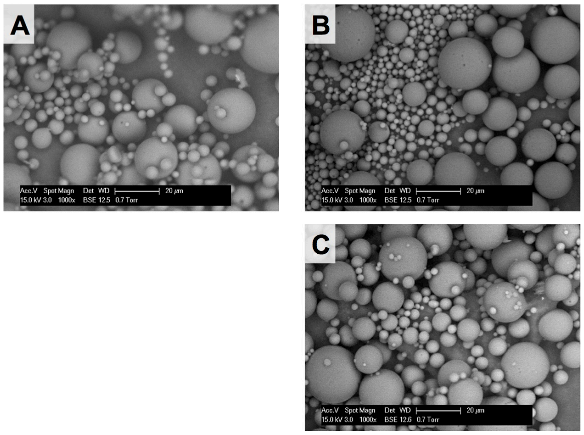

Supplementary Figure S4. IPV-containing microspheres exhibit normal morphology. Formulations F1, containing gelatin (A); F2, containing sucrose, MSG, and $MgCl_2$ (B); and F3, containing maltodextrin, MSG, and $MgCl_2$ (C) all show smooth, spherical morphology by SEM.

SUPPLEMENTARY METHODS
Production of polyclonal antibodies specific for denatured IPV

Monovalent IPV types 1, 2, and 3 (mIPV1, mIPV2, and mIPV3, respectively) were purchased from Serum Statens Institute (SSI). All antigens were denatured by incubation at 60°C for 1 hr. Polyclonal antibody production service was provided by Spring Valley Laboratories (Woodbine, MD). For each serotype, two rabbits were immunized, and serum containing polyclonal antibodies specific for IPV was recovered.

For antibody isolation, serum was filtered through glass wool to minimize lipid content and then sterilized using a 0.2-μm filter. While the serum was being stirring in a beaker, saturated ammonium sulfate (SAS) solution was added dropwise to a final concentration of 40% saturation. After 30 min of stirring at room temperature (RT), the mixture was centrifuged at 3000 rcf for 30 min at 4°C. The supernatant was discarded. The pellet was resuspended and washed using 1xPBS with 40% SAS. The pellet was once again isolated by centrifugation and the supernatant discarded. The pellet, containing antibodies, was dissolved in 1xPBS with 0.02% sodium azide. This solution was then filtered using Amicon Ultracel-15 centrifugal filters with 100 kDa MWCO in order to remove small proteins. The filtrate was discarded and the retentate washed once with 1xPBS with 0.02% sodium azide before centrifuging again. Finally, to remove more small molecules and salt contaminants, the retentate was desalted using a PD-10 column with Sephadex G-25 pre-equilibrated with 1xPBS with 0.02% sodium azide.

For each serotype, the crude antibody preparation from one of the two rabbits was chosen to be the coating antibody and was stored with 30% sterile glycerol at -80°C (long-term storage) or -20°C (short-term storage). The preparation from the other rabbit was chosen to be the detection antibody. For the detection antibody, sodium azide was not used throughout the antibody isolation process. The antibody preparation was mixed with 10-fold molar excess biotin

sulfo-NHS (N-hydroxysuccinimide) for 1 hr at RT. Unreacted biotin was removed using a PD-10 column pre-equilibrated with 1xPBS with 0.02% sodium azide. This solution of biotinylated antibodies was stored with 30% sterile glycerol as described above.

Quantification of total IPV content by ELISA

A separate assay was used for each serotype. The coating antibody preparation was diluted in 50 mM carbonate-bicarbonate buffer (pH 9.6) (type 1, 1:1000; type 2, 1:2000; type 3, 1:500), and 96-well plates were incubated at 4°C overnight with 50 μL diluted antibody per well. Unbound antibody and buffer were removed with wash buffer (1xPBS + 1% Triton X-100). All wells were blocked with 1xPBS + 1% bovine serum albumin (BSA) for 1 hr at RT. Monovalent IPV standards (from SSI) and all samples were heated to 60°C for 1 hr for denaturation, then diluted for ELISA in assay buffer (1xPBS + 1% BSA + 1% Triton X-100). Blocking solution was removed from the plates, and 50 μL of each sample or standard was added to the wells and incubated for 2 hr at RT. Unbound samples were removed using wash buffer, and 50 μL of biotinylated detection antibody preparations diluted in assay buffer (type 1, 1:2000; type 2, 1:1000; type 3, 1:2000) was added to each well and incubated for 2 hr at RT. Unbound antibody was removed with wash buffer, and 50 μL of Extravidin-peroxidase (Sigma) diluted 1:1000 in assay buffer was added to each well and incubated for 1 hr at RT. Excess reagent was removed with wash buffer. O-phenyldiamine dihydrochloride (OPD) substrate was added to each well for reaction with peroxidase, and the reaction was stopped after 30 min with 1 M sulfuric acid (H_2SO_4). Absorbance at 490 nm was measured using a Tecan multiplate

To verify that denatured IPV could be successfully measured, IPV was heat-inactivated as described above and tested using the D-antigen ELISA kit from SSI as well as the total IPV

ELISA. No signal was detected for heat-inactivated IPV using the D-antigen ELISA, while the expected concentration was detected using the total IPV ELISA.
